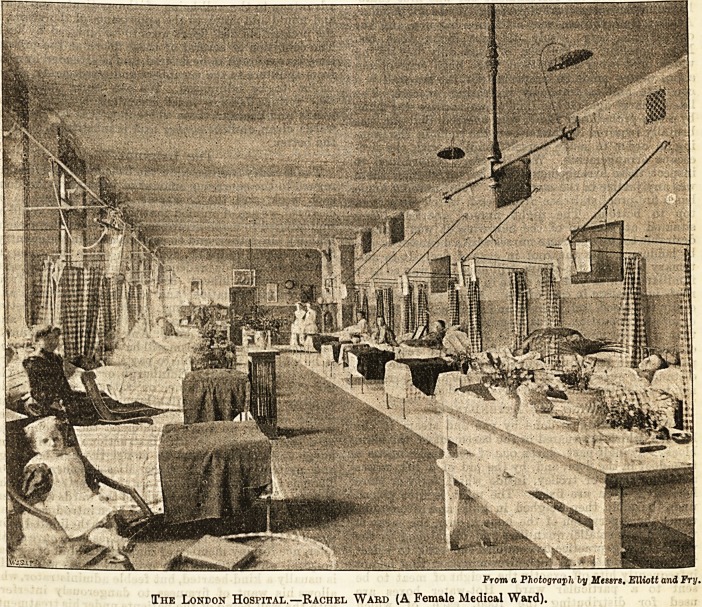# The London Hospital.—II

**Published:** 1893-06-10

**Authors:** 


### The London Hospital—II.

(*By our Special Commissioner.*)

A Hospital Lung.

It has often been said that the parks are the lungs of London. Certainly it can be said with even greater truth that the grounds at the back of the London constitute the lungs of this institution, and of its inmates. These grounds are good for the patients *per se*, because there the convalescent can gain an abundance of sun, and also, strange to say, an abundance of remarkably fresh air. Speaking of the air, an enthusiastic East-ender declared that, after all, Whitechapel is the healthiest portion of the metropolis, and on Whit Monday, beautiful day that it was, we are bound to admit that no one could desire better air or happier surroundings than those to be obtained in the London Hospital grounds in the Whitechapel Road. Apart from the immediate benefit of the air and gardens to the convalescent patient, they have an elevating effect, not only upon those patients, but upon their friends. It does the poor, who live in crowded alleys and byways, good to take a turn in the grounds, to watch the resident staff taking necessary exercise on the tennis grounds, and to see how well trees and grass can grow even in East London. As one of these visitors said with truth, when looking at the house-governor's garden at the back of his house: "Why, the Queen has not a prettier garden than that, even at Windsor!" And there was a great deal more truth in the remark than ordinary readers will credit. Why should not a movement be set on foot by Lord Meath, with the object of converting the spaces at the back of many of the tenement houses in East London into air spaces, with a few trees and seats, so that the dwellers might have some inducement to remain there, at any rate in the summer, as a substitute for the public-house? We were recently struck by accidentally finding ourselves in such a square at the back of Oxford Street, where a great number of artizans' dwellings had been put up, and where the children were happily playing about on the grass-plot, whilst their elders sat at ease under the shelter of young but growing trees. Certainly the authorities of the London Hospital have shown great wisdom in cultivating their garden ground, and we make no doubt

*From a Photograph by Messrs. Elliott and Fry.*

The London Hospital.—The Garden.

this is not the least useful of the many good works for which London has to thank them. If it be good—as it undoubtedly is—to get the maximum of sun and fresh air into the wards, surely it must be better to provide the maximum of opportunity, under the most favourable conditions, to all convalescent patients to get a thorough air and sun bath in the hospital grounds whenever the weather provides an opportunity for such treatment.

In a previous article we dealt with the work of the hospital; with the condition of the wards; the impression which the internal administration conveyed to the visitor; the vast improvements which have been introduced during the last thirty years into our hospitals; the devotion and value of the ladies in charge of the nursing; and the excellence and advantages of the open space, trees, and grass plots at the back of the hospital property. To-day we must pass on to consider other branches of the work, and we hope that such criticism as we may offer will not only be taken in good part, but will be understood to be given with a full appreciation of the continuous development which has marked the progress of the administration of the present committee, medical staff, and officers of the London Hospital.

### Special Departments.

It must be understood that the London is not only a great general hospital, but that it contains many special departments also. For instance, it has seventy-three cots in special wards for the accommodation of children under seven years of age. One great ward—"Queen"—contains fifty such cots, and we are thus able to see at a glance that in reality this is a great children's hospital, especially when we discover that very many children over seven years of age are to be found scattered throughout the hospital, few or none of the large wards being without some such cases. It follows that an intelligent donor who sends his money to the London has the satisfaction of feeling that he is not giving to one particular hospital, but to an institution which comprises the work of all hospitals, and that thus his gifts are laid out to the best advantage. There are also special departments for diseases of the eye, ear, and skin, for cancer, tumours, diseases of the bladder (including stone), and for piles and fistula. There is a maternity department, for attendance upon poor married women during confinement at their own homes within one mile around the hospital, which is placed in charge of a resident accoucheur, with two resident maternity assistants. There is also a dental department, in charge of a surgeon dentist, who attends once a week. It will thus be seen that the Committee aim at covering the whole field of medical and surgical work, a fact which has no doubt tended to materially increase the popularity of the London Hospital Medical School, and to bring it to its present high state of efficiency. There is, however, a serious defect in the organisation of these special departments, to which we desire to call attention. At the present there are only twelve beds set apart for eye diseases, and none of the other special departments have any beds allocated to them. It is true that a serious case may be sent into the wards from any one of the other special departments, but we are convinced that the sooner the Committee and medical staff made up their minds to allocate a definite number of beds for the accommodation of in-patients, under the care of the honorary officers in charge of the special departments, the better. Unless they do this their work must necessarily be incomplete, and it would be very misleading, under present conditions, to advertise that the London Hospital possesses a large number of special departments fully equipped and adequately provided with every needful appliance for the skilled treatment of all diseases. In this connection we have much pleasure in calling attention to the Clinical Records Branch, which is in the hands of two registrars, one for medical and one for surgical cases. Here are carefully preserved the history and treatment of every case admitted to the hospital for a great number of years past. The system is so excellent that is possible to refer back to the history and treatment of any given patient without delay or difficulty. The registrars also keep a full record of all pathological work done at the hospital, and the results of post-mortem examinations are carefully recorded. Clinical records like these are of the utmost importance not only to the medical school, and to the honorary staff, but to medical science at large. This branch of hospital work has received much greater attention during the last fifteen or twenty years, and the system at the London is one which is well worthy of imitation and general adoption.

### The Jewish Department.

The Committee, having to minister to the wants of a large Hebrew population, have very wisely set apart certain wards for their accommodation. The two main wards are named "The Rothschild" and "The Goldsmidt," and they form one of the most interesting portions of this unique establishment. All the food consumed by these patients is separately prepared by a Hebrew cook in a special kitchen, and everything is selected and arranged so that each patient may be served exactly as if they were in their own homes. There is also a special Jewish mortuary, which has recently been erected, and which forms one of the most tasteful and attractive buildings of the kind we have anywhere met with. It is so arranged as to enable the watcher to occupy a separate apartment outside the mortuary proper, where the duties can be adequately and reverently performed. We noticed in these Jewish wards that several of the tables, unlike those in other parts of the hospital, were without encaustic-tiled tops. The cost of converting an ordinary table in this way is from £3 to £4, and £20 would defray the cost of doing what is necessary. We are quite certain that the fact has only to be mentioned to ensure this money being sent to the secretary at once, as there are no more liberal supporters of hospitals than the members of the Jewish community. In connection with this branch of the hospital we noticed especially a separate examination room, where patients may be carefully handled by the members of the medical staff, without upsetting them or the other patients in the general wards. This is an excellent arrangement, the importance of which Professor MacEwen, of Glasgow, was one of the first to insist upon. We rejoice to notice its introduction at the London, and hope that it may soon find a place in every well-administered hospital throughout the country. We could wish that the decorations of these Jewish wards have been made as attractive as those on the floor below have been rendered through the excellent work of an amateur artist, a lady whose services deserve grateful recognition.

THE NURSES AND THE NURSING HOME.

A great point was made by the Lords' Committee of the proportion which nurses ought to bear to patients. On page 78, section 470 of that Report, the Lords' Committee give a list of the principal metropolitan hospitals, and state that "the London has 218" nurses, with a maximum number of occupied beds amounting to 733, which gives about 1 nurse to every 3⅓ patients." The importance of this statement consists in the fact that St. Bartholomew's and the Middlesex are stated to have about 1 nurse to every 3 patients, so that it follows that the staff of the London is very much too small. The fact is, however, that there are more nurses to patients at the London Hospital than at any other hospital in the whole metropolis. The Lords' Committee erroneously stated the number of occupied beds to be 733, whereas the highest number of beds fully occupied in any year, viz., 1887, was 644, and the average number occupied during the last three years has been only 616. In other words, the number of nurses to patients is considerably over 1 to 3, and not about 1 to 3⅓, as stated by the Lords' Committee. The public have heard a great deal about the food supplied to the nurses at the London Hospital. We have taken much trouble, and have made more than one inspection of the food served to the nurses, and can therefore declare, despite the critics, from actual observation, that it precisely fulfils the requirements laid down by the Lords' Committee. That Committee declared "that, from the nature of the occupation of nurses, special care ought to be exercised that, as well as being sufficient in quantity and in quality, the food should be served in an appetising manner." At the London Hospital the commissariat arrangements for nurses are as good as those at the officers' mess of a crack regiment. The meat is carved in the room, and each nurse can have exactly what she likes best, and as much of it as she requires. The vegetables, the puddings, the bread, the appointments of the table, and the service are all excellent. They could not be surpassed by the arrangements of the most careful housewife in an ordinary establishment, and every nurse can take a small bottle of beer, or stout, supplied with a patent stopper; or she can have milk and soda water, or plain water, or in hot weather, lemonade to drink with her dinner and supper. But the best test to apply to the food arrangements of an establishment is to see the people who are fed; to notice their general appearance and aspect; and to question them individually on the

*From a Photograph by Messrs. Elliott and Fry.*

THE LONDON HOSPITAL.—RACHEL WARD (A Female Medical Ward).

subject. All these tests we have applied more than once, and in the result we state with conviction that there are no happier nurses, or any better fed, or any more healthy or comfortable to be found in the world than those employed at the London Hospital.

The comforts provided in the nursing home are many and excellent. The dining and sitting rooms for nurses, and for sisters leave little or nothing to be desired. The night nurses' rooms are situated at the top of the new block where they are carefully isolated, so as to be protected from noise or disturbance of any kind. In order to ensure the maximum of comfort to the nurses a special pantry has been provided in the home, where any nurse can obtain boiling water for her teapot at any hour of the day or night. Talking of teapots reminds us of a little practical point which is well worth recording. When the refreshment bar was first established in the out-patient department it was not popular. At that time the tea was made in a big urn, and the out-patients very wisely did not drink that tea. Now, however, as the urn is used only for boiling water, and every out-patient who likes can obtain a cup of tea made in a special pot, the tea-stall is a complete and continuously increasing success. The latest charge against the London authorities was made by a late probationer nurse, who averred that she was brutally required to wander over the roofs in the evening, in order to make herself familiar with the fire-escape arrangements. We, therefore, determined to inspect these arrangements, and to see whether there was any justice in the complaint. The nursing home has a fire-proof staircase at one end, which runs from top to bottom. The night nurses' quarters, being situated at the top of the new wing, it was thought desirable to provide these nurses with a means of escape by ladders across the roof in case of fire. It is customary to have a fire-escape drill once in a way so as to familiarise them with the route to be taken across the roofs in question in case of fire. These roofs are wide, easily traversed, well lighted, and supplied with ladders of ample proportion. Our inspection has convinced us that a nurse might decline to do the fire-drill from obstinacy, or for some other reason, but no reasonable person would for one moment object to traverse the short distance in question with the aid of the ample arrangements which the authorities have provided.

### Cooking and Attendance.

All the cooking is done by gas and steam in the kitchen, which is situated in the basement of the main block. At half-past eleven one of the cooks opens a trap-door in the floor, and, by the aid of a lift, raises an iron frame or trolley, inside which forty or more legs of mutton are fixed. The meat is first placed upon the table, then weighed in bulk, and afterwards, under the supervision of the housekeeper, the meat is weighed into smaller quantities and dispatched to the wards. Long practice has enabled the cook to select the cooked joints with such discrimination that he not infrequently hits off exactly the weight of meat to be sent to a particular ward. Hot-water boxes are used for distributing the food, each of which bears the name of the ward to which it belongs. With the aid of the diet-sheet, not only the meat, but the fish, both boiled and fried, vegetables, puddings, chops, and other diets are duly sorted out, and the whole are sent from the kitchen within half-an-hour from the time that the dinner commences to be served. The beef-tea is distributed in cans, and consists of three kinds: in the preparation of one sort eight ounces of beef is used to the pint; in another sixteen ounces; and for extra strong beef-tea for special cases three pounds of meat to the pint is the rule. Every pint of the last kind of beef-tea costs half-a-crown. Some idea of the cost of feeding the patients and staff of the London Hospital Hospital may be gathered from the statement that, during 1892, £6,589 was spent upon meat; £1,279 upon bread; £718 upon vegetables; £2,753 upon milk; £1,302 upon eggs, and £1,528 upon butter and cheese; fish cost £1,403, and groceries £1.443. The total cost for provisioning the London Hospital for twelve months amounted altogether to £17,961, exclusive of £730 spent on wines, spirits, and beer as medicine. It is useful perhaps to quote these figures, because they will bring home, to the minds of all housekeepers at any rate, the vastness of the work done at Whitechapel.

### Defects Still to be Remedied.

In addition to the desirability of removing the patients from the few remaining basement wards at present in use, there are one or two other things which the wealthy might usefully aid the Committee to accomplish. First of all, it would be an immense advantage to the well-being of the patients and staff, and would add much to the appearance of the wards, if the present old deal floors were to be replaced by teak. The condition of several of these floors at the present time leaves much to be desired, and is attended by risks from splinters to the scrubbers and ward-maids, which ought to be minimised. We can quite understand that serious damage must sometimes be done to the hands and knees of those whose duty it is to keep the wards clean, and the sooner teak is substituted for deal the better.

### The Laundry.

The laundry is situated in the basement, and, having regard to the enormous amount of washing to be done each week, and the character of the clothes to be cleansed, we are of opinion that the Committee would be well advised to make arrangements to close the present laundry altogether, and to establish one in the country. Such a step is desirable, not only because the room at present available is inadequate to the work, but because of the unhealthiness calculated to arise from its present situation and confined position, and also because the washing could be much better and more cheaply done if the machinery and appliances were up-to-date. Any governor who doubts the justice of this criticism will have all doubts removed if he will take the trouble to pay a visit to the laundry at the Royal Infirmary, Edinburgh, or Glasgow. Should the Committee determine, or rather, should some wealthy governor or philanthropist enable the Committee to determine to remove the present laundries to the country, we would recommend the appointment of a small committee, with instructions to visit Edinburgh and Glasgow, and to model the new buildings and system upon that which Miss Spencer and Dr. Thomas have brought to such a state of perfection in the North. We observed that some of the wards were overcrowded, and that extra beds had been introduced to an extent which dangerously interfered with the limited floor-space available. Our experience teaches us to have small tolerance for any member of the medical staff of a great hospital who systematically overcrowds his wards. He is usually a kind-hearted, but feeble administrator, who allows his want of firmness to dangerously interfere with the well-being of the patients under his treatment, and possibly to endanger also the health of the whole hospital. We hope that house-visitors, and members of the Committee, will set their faces against the introduction of extra beds into any ward of the London Hospital, seeing that, from the age of the buildings, it is not safe to increase the number of beds beyond the limit fixed as a maximum, after careful consideration of the floor and air space available. There are spots on the sun, and these defects, and the relatively few criticisms we have made, only tend to bring out the uniform excellence of the administration of this great institution. It is a marvellous establishment in many ways, and the quality and character of its work can bear comparison with that of any similar institution in the world.

## Figures and Tables

**Figure f1:**
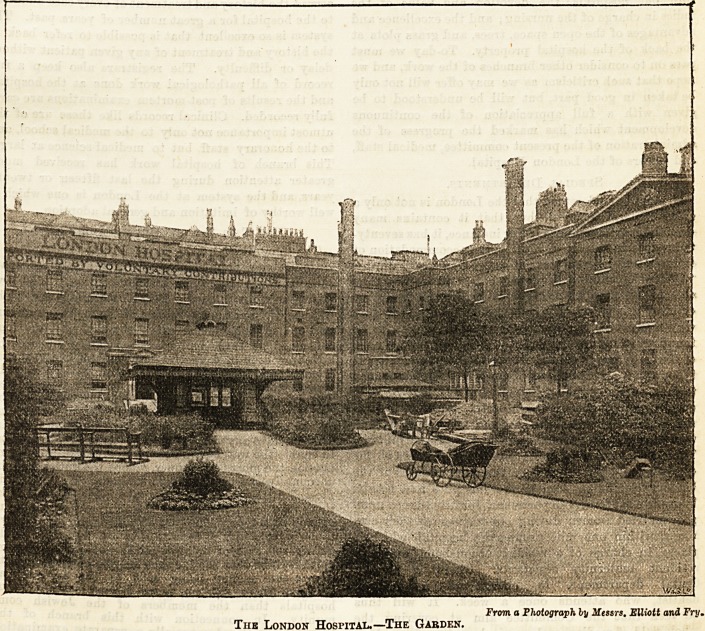


**Figure f2:**